# Benign tumors broaden the field of application for immunotherapy

**DOI:** 10.3389/fimmu.2025.1593960

**Published:** 2025-06-24

**Authors:** Mohamed A. Youssef, Hisham Al-Sharif, Brian T. McGrath, Maria M. Picken, I. Caroline Le Poole

**Affiliations:** ^1^ Department of Internal Medicine, University of Texas Medical Branch, Galveston, TX, United States; ^2^ Department of Pathology, The University of Texas Medical Branch, Galveston, TX, United States; ^3^ Department of Medical Oncology, Ohio State University, Columbus, OH, United States; ^4^ Departments of Dermatology, Microbiology and Immunology, Robert H. Lurie Comprehensive Cancer Center, North Western University, Chicago, IL, United States; ^5^ Department of Pathology, Loyola University Medical Center, Maywood, IL, United States

**Keywords:** benign tumors, mutations, antigens, immunotherapy, checkpoint inhibitors, tumor vaccines, adoptive T cell therapy

## Abstract

Immunotherapy has shown significant potential for treating malignancies. Not yet widely considered is the opportunity to employ immunotherapy for the treatment of benign tumors. By focusing on targetable antigens expressed following specific genetic changes associated with individual benign tumors, immunotherapy may provide an effective approach to benign tumor treatment, circumventing the need for more conventional surgery. Immunotherapies can specifically recognize and target tumor cells, which could be especially beneficial for benign tumors given the extended timeframe available for treatment. Thus, benign tumors, offering a greater window of opportunity for treatment and a relatively stable phenotype associated with a limited mutation burden, can derive great benefit from immunotherapeutic approaches targeting antigens uniquely associated with each condition.

## Graphical Abstract

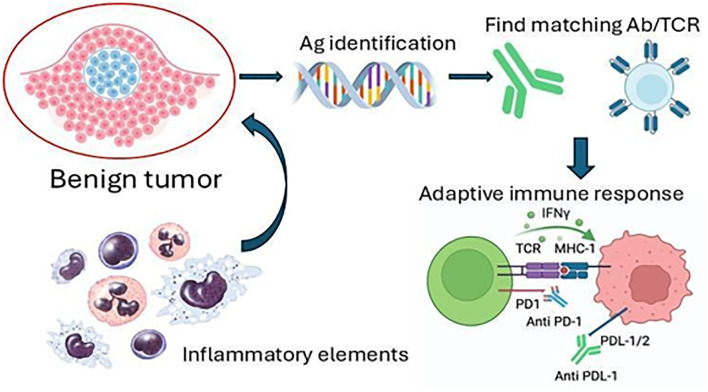

## Introduction

Neoplastic somatic cells form benign or malignant tumors in diverse organs. Benign tumors are typically non- invasive and well-circumscribed compared to more aggressive malignant tumors. While benign tumors generally do not present an acute risk to life, they considerably affect an individual’s quality of life by manifesting various symptoms and complications. For example, they may provoke an overproduction of hormones, causing systemic side effects such as hypertension. Other benign tumors are more directly life-threatening due to their location. Intracranial benign tumors, for example, can disrupt vital functions such as breathing by their growth in the brainstem and other critical areas (1). When left unaddressed, the progression of benign tumors can cause compression of nearby structures or potentially fatal obstruction of blood and lymph flow (2).

We present benign tumors as potential targets for immunotherapy, expanding upon the concept of our prior studies with transgenic T cells for the treatment of lymphangioleiomyomatosis (LAM) and tuberous sclerosis complex (TSC) (3). While immunotherapy has achieved remarkable successes in treating malignancies including melanoma and non-small cell lung carcinoma, its application to benign tumors remains underexplored. Historically, benign tumors have been considered less impactful for patient quality of life, prompting limited therapeutic innovation. However, some benign tumor patients face unmet medical needs, making them strong candidates for immunotherapy. This review examines the risk-benefit profile of these approaches and identifies benign tumor subtypes with the greatest potential for therapeutic intervention. As potential therapeutic approaches, we considered currently approved therapies such as immune checkpoint inhibitors (ICI), transgenic T cells and next generation modalities including personalized mRNA vaccines (4).

We recognize the importance of considering combinatorial approaches to establish lasting responses (5). Ongoing innovation continues to bring new platforms, engineered safety mechanisms, and increasingly effective strategies to be tailored for benign tumor conditions. This progress is reflected in strategies such as suicide genes inserted in the constructs used for T cell transduction in order to eliminate the therapeutic cells as needed, including cytokine genes to sustain transgenic T cell activity, combining anti-PD1 with transgenic T cells to prevent their premature exhaustion, preceding checkpoint inhibition by anti-tumor vaccination and other combined measures (6).

Treating benign tumors by immunotherapy presents compelling advantages. Unlike malignancies, benign tumors are relatively slow-growing with limited metastatic activity, offering a broader window of opportunity and more localized areas for treatment. The extended time frame allows for careful adaptation of immunotherapy strategies, maximizing the potential for sustained responses. Benign tumors are frequently associated with chronic inflammation, suggesting recognition by the immune system (7). However, ineffective development of full adaptive immunity ultimately leads to disease progression. Like malignancies, benign tumors result from specific genetic alterations. Cancer-driving mutations provide malignant tumors the ability to invade and metastasize, and mutations in similar genes exist in benign tumors (8). Mutations linked to benign tumor conditions are more consistent and the corresponding disrupted biology can be used to identify tumor- specific antigens. While generic immune approaches including immune checkpoint inhibitors are used, it must be recognized that mutations are relatively sparse in benign tumors, resulting in limited specificity and efficacy. The tumor specific antigens associated with individual benign tumor types meanwhile offer steady targets for directed approaches. A critical factor in the efficacy of immune-based interventions is the immune-tumor interaction. The malignant tumor microenvironment becomes increasingly immunosuppressive, which can render immunotherapies ineffective (9). Benign tumors are more likely to be responsive to immunotherapeutics, especially when antigens of premalignancy are identified to guide adaptive responses and local inflammation can be overcome (10). Conventional approaches to benign tumor treatment include surgery, radiation and targeted chemotherapy. Some limitations of these approaches include disfigurement and infection risk, the possibility of introducing mutations that facilitate malignant transformation and halting progression rather than mediating tumor elimination. These current benign tumor treatments can bring extreme physical and emotional stress.

Immunotherapy has become a transformative development in oncology. Meanwhile, current benign tumor treatments, including surgery and hormone treatments, can bring extreme physical and emotional stress, and the possibility of a recurrence after treatment is significant. Early introduction of immunotherapy could facilitate limiting the reliance on invasive procedures to safeguard the quality of life. These factors provide a rationale for considering immunotherapy as a treatment option for benign tumors.

## Methods

An extensive literature review was conducted to explore therapeutic approaches for benign tumors, encompassing scientific studies, clinical trials, and reviews focused on promoting adaptive immune responses to benign tumors. Data sources included peer-reviewed journals, clinical trial registries, and medical databases. Research involved identifying and analyzing mutated genes associated with benign tumors; using this genetic information, strategies were derived from methodologies and treatments used for other conditions. Understanding the molecular background of benign tumors is important to predict the resulting physiologic changes in specific tumor types. Existing therapies were reviewed, but the primary focus was on developing novel approaches tailored to the identified genetic alterations to expand the current therapeutic landscape. Summarizing our findings, **Tables 1**, **2** provide common benign tumor types, their symptoms, current treatment options, mutations that drive these tumors, and the current treatment options.

**Table 1:** Disease characteristics and potential targets of immunotherapies.

Tumor Type	Prevalence	Symptoms	Diagnosis	Treatment	Cell Type	Genetic Alterations/Mutations	GPossible Targets for Immunotherapy
Adenomas	Adenomatous polyps: ~30.2%, increases with age, particularly after age 65 (11) FAP affects 1 in 11,300-37,600 individuals (11) Pituitary adenomas is ~1 in 1000 adults (12) Adrenocorticotr opic hormone (ACTH)- secreting tumors account for 2% to 6% of all adenomas (12)	FAP: rectal bleeding, anemia, alternating diarrhea and constipation (11) Pituitary adenoma: delayed puberty, growth retardation, visual field reductions. Functional hormone imbalance, with prolactinomas being the most common (12)	Endoscopy MRI/CT Scan Sonography (13)	Surgery, ablation, somatostatin analogs, manipulating tumor hormone secretion, cabergoline, pegvisomant (13)	Colonic Adenoma: Glandular colon epithelial cells (14) Pituitary Adenomas: lactotrophs, somatotrophs, corticotrophs, thyrotrophs, gonadotrophs, cells (15)	Familial adenomatous polyposis: *APC* (adenomatous polyposis coli) (13) Adrenocortical tumors: *CTNNB1*, *PRKACA* , and *KCNJ5* (16)	Colorectal adenomas: MUC1 peptide vaccine, Truncated APC protein (TASIN-1), MMP9 (13, 17) Pituitary adenomas: GNAS- mutated protein, PD-L1, DNAJB1-PRKACA fusion neoantigen (16)
Leiomyomas	~75% of women over 50 present with fibroids (18)	Pelvic pressure, pain, menorrhagia, dyspareunia, constipation, urinary symptoms, reproductive issues (19)	Ultrasound Sonohysterography MRI Hysteroscopy (19, 20)	Gonadotropin- releasing hormone agonists, progesterone receptor modulators, high-intensity focused ultrasound (HIFU), uterine artery embolization (UAE), surgery (myomectomies and hysterectomies (19, 20)	Spindle-shaped smooth muscle cells separated by collagen fibers (19)	MED12 (mediator complex subunit 12), HMGA2 (high mobility group AT- hook 2), FH (fumarate hydratase) COL4A5 and COL4A6 (21)	MED12-mutated protein, Wnt/β-catenin pathway components (21)
Hemangiomas	Infantile cutaneous hemangiomas ICHs): ~4-10% (22)	A red to reddish-purple raised growth up to 2 inches in size on the skin with visible blood vessels (23)	Physical exam MRI/CT (23)	Beta-blocker medicines, corticosteroid medications, laser surgery (23)	Vascular Endothelial cells (24)	Infantile hemangiomas: *VEGFR* (23) Cutaneous hemangiomas: *GNA14*, *GNA11*, *GNAQ*, *IDH1* and *IDH2* (25)	VEGFR2, PD-1/PD-L1 pathway, GNAQ/GNA11 Q209L/P mutant proteins (25)
Meningiomas	~53 per 100,000 individuals, 30% of primary intracranial tumors (26)	Visual disturbance, headache, hearing loss, memory loss, loss of smell, seizures, weakness in arms or legs, language disorder (27)	MRI/CT (28)	Surgery, ablation, radiotherapy (28)	Arachnoid cap cells, neural crest-derived (26)	*TRAF7, KLF4, AKT1*, and *SMO* (29) Higher-grade meningiomas located in the cerebral and cerebellar hemispheres: *NF2* (29)	PD-L1, WT1 protein, CCR2, CD163 (29)
Melanocytic Nevi	Congenital melanocytic nevi (CMNs): global prevalence of 0.2% to 6% (30, 30) Acquired melanocytic nevi: 7% of children (30, 31)	Asymmetric lesions, border irregularity, color variegation (32)	Dermatoscopy skin imaging/ Total body photography biopsy (33)	Shave removal surgical excision, laser therapy, cryotherapy (33)	Melanocytes in the epidermis (33)	UV-induced: *BRAF* Congenital: *NRAS* (32)	NRAS Q61K/R neoantigen peptides, Melanosomal proteins (MART-1, tyrosinase, gp100) (34)
Neuromas	Symptomatic neuroma after nerve injury: 5-10% (35) Symptomatic neuroma after amputation: 7- 25% (35) Vestibular schwannomas: 6% of intracranial tumors (36)	Symptomatic neuroma: neuropathic pain, allodynia, cold intolerance, hypesthesia, dysesthesia (37) Vestibular schwannoma: hearing loss, tinnitus, vertigo, facial numbness (36)	Symptomatic neuroma: patient history, physical examination, response to diagnostic nerve block, X-ray/MRI (35) Vestibular schwannomas: CT/MRI (36)	Symptomatic neuroma: targeted muscle reinnervation, desensitization therapy, anesthetic injections, antidepressants, anticonvulsants, opioids (38, 39) Vestibular schwannomas: observation, surgery, gamma knife radiosurgery (36)	Symptomatic neuroma: disorganized nerve fiber bundles, fibrous stroma, Schwann cells, perineural cells, axons and endoneurial fibroblasts (35) Vestibular schwannomas: Schwann cells, connective tissue, and axons (40)	Vestibular schwannomas and sporadic forms: *NF2* (41)	Symptomatic neuroma: CGRP, IL-6, Foxp3+ T-regs, M2 macrophages (37) Vestibular schwannomas: NF2-mutated protein, VISG4, HLA-DPB1, VEGFR (42)
Osteochond- romas	1-3% of the total population (43)	A painless hard mass below-normal- height, differential limb lengths, pressure or irritation with exercise, soreness of nearby muscles (44)	X-ray, CT/MRI (44)	Surgery (44)	Chondrocytes (45)	EXT1/EXT2 (46)	BMP signaling pathway components (46)
Condylomas	Worldwide prevalence of HPV infection in women without cervical abnormalities: 11-12% (47)	Benign growth in various body parts, sometimes bleeding and cancer formation (48)	Histopathology of lesions primarily detection of viral DNA, polymerase chain reaction (PCR) Southern Blot (48)	Excision surgery, cryotherapy (48)	Squamous epithelial cells (49)	Head and neck papillomas: H- RAS, K-RAS ( 50)	HPV6/11 E7 protein, PD-L1 (51)
Lipomas	1-2% of the total population (52)	Fatty lump between the skin and the underlying muscle layer (52) Dercum's disease: painful fatty growths, brain fog, fatigue, weakness, palpitations, anxiety, cardiac arrhythmia, gastrointestinal issues (53)	Histopathology of lesions primarily detection of viral DNA, polymerase chain reaction (PCR) Southern Blot (48) Clinical examination, CT/MRI (54)	Excision surgery, cryotherapy (48) Excision surgery, liposuction (52)	Squamous epithelial cells (49) Adipocytes (52)	Head and neck papillomas: H- RAS, K-RAS ( 50) Lipomas: HMGA-2 (55) Dercum's disease: unknown.	HPV6/11 E7 protein, PD-L1 (51) CD19 CAR therapy (55)

**Table 2:** Ongoing preclinical and clinical studies.

Benign Tumor Targeted	Immunotherapy or Vaccine	Clinical Stage	Target	Population and intervention	Outcomes	Future Implications
Colorectal adenomas	MUC1 peptide vaccine (17)	Phase I/II (17)	*MUC1* *(* 13) (17)	Population: individuals aged 40- 70 years old diagnosed with advanced adenoma within less than one year from a clinical trial (17) Intervention: MUC1 peptide vaccine at 0, 2, and 10 weeks then booster dose at 53 weeks.	A 38% reduction in recurrence compared to placebo (17)	Combining the MUC1 vaccine with immunomodulatory therapies to counteract suppressive cells like MDSCs, potentially enhancing vaccine efficacy (17, 56).
Pituitary adenomas	Checkpoint inhibitors (e.g., Anti-PD-1/PD- L1) (57)	Preclinical (57)	*PD-L1* *(* 57)	Targeting low PD- L1 expression with checkpoint inhibitors (57)	Potential for targeted therapy in specific adenoma subtypes (57)	Further research into checkpoint inhibitors for treating aggressive and invasive adenomas with low PD-L1 expression (58)
Leiomyomas	Checkpoint inhibitors (e.g., Anti-PD-1/PD-L1), MED12-Targeted vaccines (59)	Preclinical (59)	*MED12* (59)	MED12 mutations in uterine fibroids linked to abnormal estrogen signaling and tumor development (59)	Potential immunotherapeutic targets identified (59)	Checkpoint inhibitors and MED12-targeted vaccines could offer new treatments, particularly in cases where the immune environment is suppressed by MED12 mutations (59)
Hemangiomas	PD-1 inhibitors (25)	Preclinical (25)	*PD-1* (25)	Use of immune checkpoint inhibitors targeting PD-1 in treating hemangiomas (25)	Early outcomes show promising reduction in size and progression (25)	Further clinical trials to establish the efficacy and safety of PD-1 inhibitors in hemangioma treatment (25)
Meningiomas	WT1-specific T cells (60)	Preclinical (60)	*WT1* (60)	WT1-targeted adoptive immunotherapy in malignant skull base meningiomas (60)	Significant tumor growth inhibition and prolonged survival in a mouse model (60)	Potential for clinical trials in humans to evaluate the effectiveness of WT1-targeted therapies (60) Checkpoint inhibitors combined with WT1-specific T cells enhance tumor targeting by boosting immune response and preventing immune suppression (60)
Melanocytic Nevi	PD-1 inhibitors, Adoptive T-cell therapy targeting MART-1 (61)	Preclinical (62)	*BRAF*, *NRAS*, *MART-1* (61)	Use of immune checkpoint inhibitors Pembrolizumab and Ipilimumab to treat nevi and associated melanomas (62)	Regression of nevi and melanoma lesions in trials (63)	Long-term outcomes of checkpoint inhibitors in benign nevi treatment. Exploring MART-1 and other melanosomal proteins as safe targets (63) In UV-induced tumors, Vemurafenib might precede ICI therapy.
Vestibular Schwannoma	VEGFR peptide vaccine (64)	Phase I/II (64)	VEGFR1, VEGFR2 (64)	Patients with progressive NF2- derived schwannomas (64)	Hearing improvements, tumor volume reduction, VEGFR1 & VEGFR2-specific Cytotoxic T lymphocytes (64)	Potential for novel peptide vaccine which reduces tumor size and improves hearing loss (64)
Osteochond- romas	RARγ agonists, T- cell targeting ERK pathways BMP signaling antagonists (65)	Preclinical (65)	EXT1, EXT2, ERK1/2 (65)	Inhibiting osteochondroma development through RARγ agonists (65)	Inhibition of tumor growth and modulation of cartilage homeostasis. Novel mutation (c.1173 + 2T>A) in the ext2 gene, causing hereditary multiple exostoses (65)	Investigating RARγ agonists and ERK pathway targeting in clinical trials for osteochondromas, potentially in combination with immunotherapy (65)
				Systemic treatment with BMP signaling antagonists in mouse models to reduce osteochondroma Formation (65)		Clinical trials to explore the use of BMP antagonists and other targeted therapies in humans.
Condylomas	Therapeutic HPV vaccines, Checkpoint inhibitors (e.g., Anti-PD-1/PD-L1) (51)	Approved, Preclinical (51)	HPV (51)	Vaccination to prevent and treat papilloma formation, particularly in high-risk populations (51)	Prevention of cervical cancer Significant reduction in genital warts (51)	Expand vaccine to target a wider array of viruses, as other subsets can replace subtypes 16 and 18 (51) Expanding therapeutic vaccines and checkpoint inhibitor applications in HPV-related papillomas (51)
Lipomas	CAR T cell therapy (55)	Preclinical (55)	*HMGA2* *(* 55)	Adoptive T-cell therapy targeting HMGA2 in lipomas (55)	Improved anti-tumor immunity and potential for reduced tumor size (55)	Design of peptide vaccines to promote immune targeting (55)

### Adenomas

#### Familial adenomatous polyposis

Adenomas commonly develop from glandular tissues and their clinical presentations vary based on organ location and on specific glandular functions (14). Nearly all FAP patients progress to colorectal cancer if left untreated (11, 14). Pathogenic variants of benign adenomas provide insight into tumor pathogenesis. FAP diagnosis involves endoscopic examination and genetic screening for mutated Adenomatous Polyposis Coli (*APC*), a multifunctional tumor suppressor gene, and *MUTYH*, a base-excision repair gene protecting against DNA damage from oxidative stress; germline mutations in *APC* are present in 80% of FAP patients (13). Tumor recurrence is common within the first year of follow-up, involving new lesions, *de novo* mutations, or remnants from incomplete resection (13).

Immune prevention strategies have been explored for adenomas. MUC1 glycoprotein was identified as a tumor associated antigen for gastric cancers (66) In a phase II study, most patients with advanced colorectal adenomas treated with a MUC1 vaccine plus adjuvant poly-ICLC (polyinosinic-polycytidylic acid, carboxymethylcellulose, and poly-L-lysine) exhibited significant anti-MUC1 IgG antibody titers, sustained responses and a 38% reduced adenoma recurrence (17) Alternatively, three bispecific antibodies (bsAbs) for bile duct carcinoma (BDC) immunotherapy: anti-MUC1 x anti-CD3 (M x 3), anti-MUC1 x anti-CD28 (M x 28), and anti-MUC1 x anti-CD2 (M x 2) were developed (67). Combining all three bsAbs together with T lymphokine-activated killer cells (T-LAK) cells rendered the greatest tumor- killing efficacy *in vitro* in BDC-grafted mice, indicating a potential treatment strategy. This could mark a significant shift in the management of FAP and offer a preventive approach to complement or replace surgery.

#### Pituitary adenomas

**Figure 1:**
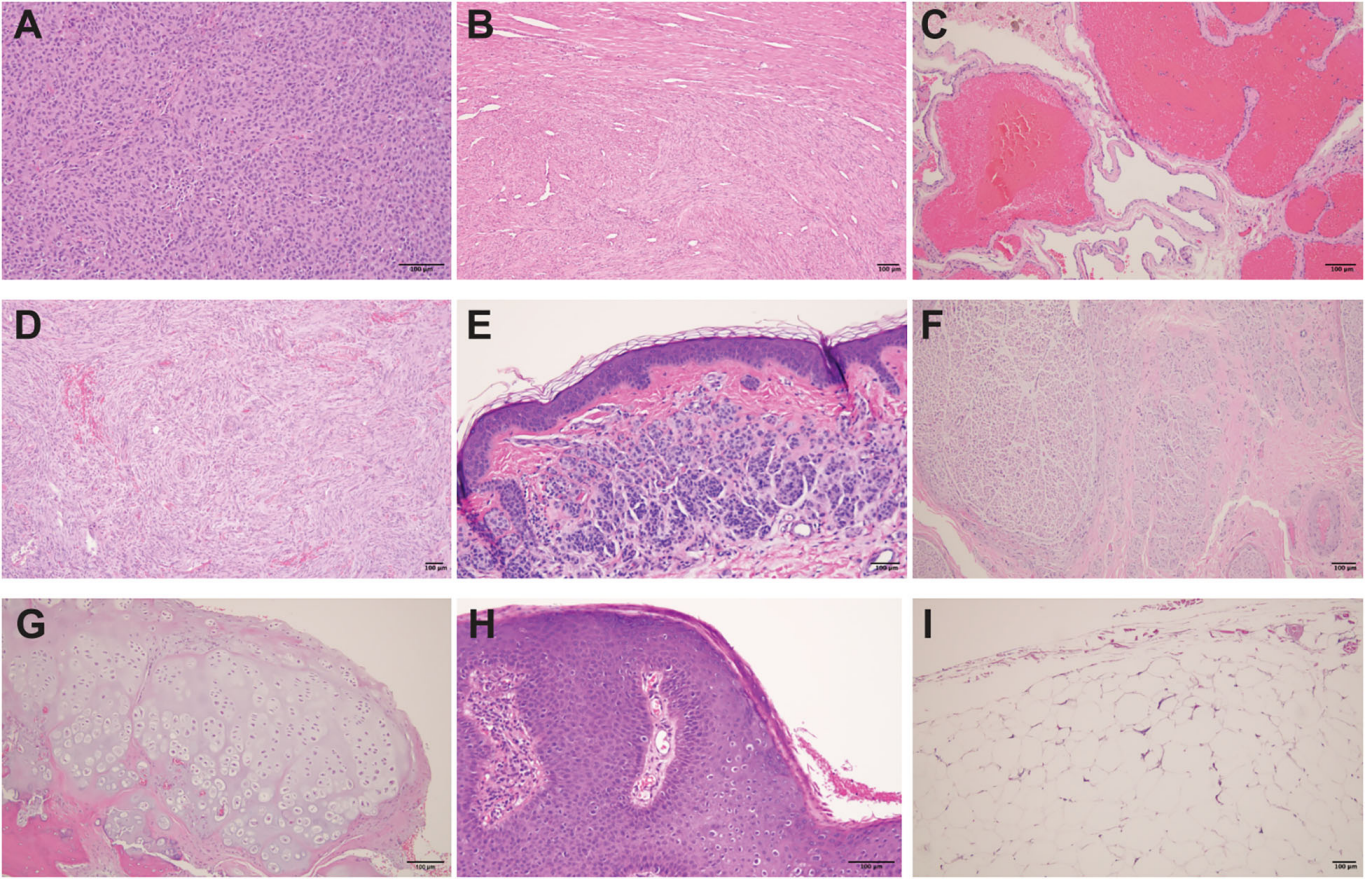
Histological features of various benign tumors and lesions. **(A)** Pituitary neuroendocrine tumor (pituitary adenoma): Solid sheets to nests of epithelioid cells with abundant acidophilic cytoplasm within a fibrovascular stroma. **(B)** Uterine leiomyoma (fibroid): Benign tumor of smooth muscle origin, composed of intersecting fascicles of spindle-shaped cells with indistinct borders, eosinophilic cytoplasm, and cigar-shaped nuclei. **(C)** Section of liver with cavernous hemangioma: Benign vascular tumor composed of variably sized, dilated and thin- walled vessels lined by a single layer of flat endothelial cells. **(D)** Meningioma: Benign neoplasm of cerebral meninges composed of spindle cells with meningothelial whorls and psammoma bodies. **(E)** Acquired intradermal melanocytic nevus: Nested proliferation of melanocytes in the dermis. The cells have scant cytoplasm, regular nuclei and are separated by a collagenous stroma. **(F)** Neuroma: Disorganized spindle cell proliferation of nerve components. **(G)** Osteochondroma: Mature hyaline cartilage with overlying fibrous perichondrium. **(H)** Condyloma acuminatum: Hyperplastic papillomatous squamous proliferation with a fibrovascular core and focal koilocytosis. **(I)** Lipoma: Benign soft tissue tumors characterized by uniform proliferation of mature adipose tissue. All images represent H&E (hematoxylin and eosin) stained tissue sections of paraffin-embedded tissues, showing the physiologic variety to indicate that benign tumors might be selectively targeted.

Pituitary adenomas (**Figure 1A**) arise from the anterior pituitary gland (12) from either gonadotropin-producing cells, CD15+ cells, or tumor stem-like cells (15). These cellular origins exhibit unique stem cell gene expression profiles, growth behavior and hormone secretory activity. Some variants display a more aggressive clinical behavior with higher recurrence rates and resistance to standard therapies. These include sparsely granulated somatotroph adenomas, silent corticotroph adenomas, Crooke’s cell adenomas, and immature PIT1-lineage adenomas (15).

Pituitary adenomas are triggered by somatic mutations that disrupt intra-pituitary signaling and promote benign cell proliferation (12) Mutations in genes such as *ACAsCTNNB1, PRKACA*, and *KCNJ5* underlie adrenocortical tumors (16)*. GNAS* (guanine nucleotide- binding protein alpha subunit) mutations, particularly at R201 and Q227, are linked to uncontrolled cAMP accumulation and cellular proliferation in approximately 35% of functioning pituitary adenomas (58). Constitutive activation of the Gsα protein results in continuous stimulation of adenylate cyclase, causing elevated cyclic AMP (cAMP) and cellular proliferation. Vaccines have been developed to target the mutated Gsα protein, eliciting an immune response (68). Early studies show promise, particularly when combined with immune checkpoint inhibitors (57). Therapeutics impacting downstream signaling molecules within the cAMP/PKA/CREB pathway could further improve treatment outcomes (58). High invasiveness of pituitary adenoma subtypes are correlated with low expression of *MSH6/2* and *PD-L1*, and early intervention is key.

CTAG2 and TSPYL6 nonfunctional pituitary adenomas show promise for immunotherapy, with studies suggesting benefits including CD8+ T cell infiltration (69). Counter-intuitively, the presence of tumor-infiltrating lymphocytes (TILs) in pituitary adenomas is linked to immunosuppression and recurrence, with significantly worse post-surgery outcomes; 61% of patients with TILs experience tumor persistence or recurrence involving increased immune suppression by regulatory T cells (Tregs) and derivative cytokines (70). Thus, targeting immunosuppressive components among TILs might provide a therapeutic avenue for pituitary adenoma patients. Moreover, tumor-associated macrophages (TAMs) in pituitary adenomas are correlated with increased tumor growth (71). Targeting MMP9, overexpressed in CTNNB1- mutant hepatocellular carcinoma, restored CD8+ T cell function and enhanced anti-PD-1 efficacy (72). We therefore suggest a strategy targeting MMP9 in adrenocortical adenomas with CTNNB1 mutations.

### Leiomyoma (fibroma)

Leiomyomas (**Figure 1B**) primarily consist of smooth muscle growth and seventy % of leiomyomas are driven by mutations in *MED12* (**Table 2**). MED12 helps the multiprotein Mediator complex regulate transcription (18). Pathogenic *MED12* mutations disrupt interactions with the cyclin C-CDK8/CDK19 complex, leading to upregulated estrogen signaling and leiomyoma development (21). *MED12* mutations activate the Wnt pathway, potentially cooperating with estrogen in leiomyoma development. *MED12* mutations and *HMGA2* gene rearrangements represent two mutually exclusive pathways in leiomyoma development, with *MED12*-mutated tumors typically being smaller but more numerous (21, 73). Beyond initiating fibroid development, *MED12* mutations are also associated with immune suppression within fibromas, rendering them potential targets for immunotherapy to enhance immune cell infiltration and activation. This alternative to surgery potentially enables long-term management (59).

### Hemangiomas

Hemangiomas are common neoplasms of proliferating vascular endothelial cells (24) and are classified as capillary, cavernous (**Figure 1C**), tufted, or mixed based on their histology. With an incidence of 10%, hemangiomas are divided into congenital hemangiomas or infantile hemangiomas (IH) (22) Though most infantile hemangiomas self-resolve by age 9, up to 8% may cause cosmetic issues and require treatment (22). While most hemangiomas occur sporadically, IH was linked to chromosome 5q31-33, containing candidate genes FGFR4, PDGFR-β, and FLT4 (23). These genes are involved in blood vessel growth, angiogenesis, VEGF signaling and MAPK regulation pathways.

The immune environment of infantile hemangiomas is marked by gross overrepresentation of CD11b+ dendritic cells that contribute to tumor development and immunity (74). Activated dendritic cells interact with CD4+ T cells, including regulatory T cells, releasing proangiogenic VEGF, IL-6, TNF-α, and IL-8. These support endothelial cell survival and promote hemangioma stem cell (HemSC) proliferation (75). CD163- expressing M2-polarized macrophages that express DC-SIGN further stimulate angiogenesis, inflammatory responses, and IH growth (76).

Checkpoint inhibitors, adoptive cell transfer, and vaccine-based therapies offer promise for the treatment of hemangiomas (**Table 3**). Prominent PD-1 expression by TILs is a sign of T cell activation in IH (80). VEGFR2 and endoglin (CD105) are highly expressed in proliferating IH, providing distinct opportunities for immunotherapy (75). Overexpressed on hemangioma cells, they could be targets for vaccines that ‘educate’ the immune system. A phase 1 glioblastoma study revealed that combining a VEGFR-2 DNA vaccine and anti-PD1 generated safe and detectable immune responses to VEGFR-2 (25). T cells might specifically eliminate hemangioma cells displaying these antigens. These immunotherapeutic strategies could provide new treatments for hemangiomas, especially those resistant to conventional therapies.

**Table 3:** Potential target antigens for TCR and CAR transgenic T cells compared with other immunotherapeutic agents.

	Adoptive T-Cell Therapy (TCR/CAR-T Cells) (5, 33, 60, 77, 78)	Checkpoint Inhibitors (PD-1, CTLA-4) (5, 57, 79)	Tumor Vaccines (5, 9, 17, 33, 34, 57, 74)
Mechanism of Action	TCR and CAR-T cells are engineered to specifically recognize and attack certain antigens on the surface of tumor cells, leading to their direct elimination.	They enhance the immune response by blocking inhibitory signals (PD-1, CTLA-4) that prevent T cells from attacking cancer cells, thereby reactivating the immune system to target the tumor.	Training the immune system to recognize and attack cancer cells through specific tumor- associated antigens.
Potential Targets in Benign Tumors	- Meningiomas: WT1 - Hemangiomas: VEGFR2, Endoglin (CD105) - Melanocytic nevi: MART-1, BRAF, NRAS mutations - Lipoma: HMGA2-expressing cells.	- Hemangiomas: High PD-1 expression - Meningiomas: PD-L1, B7-H3 - Papillomas: PD-L1 in recurrent respiratory papillomatosis (RRP) - Melanocytic nevi: PD-1 in NRAS-mutated nevi	- Colorectal adenomas: MUC1 peptide - Meningiomas: WT1 - Melanocytic nevi: MART-1, Tyrosinase
Safety Profile	- Potential for severe side effects such as cytokine release syndrome (CRS) and neurotoxicity - Risk of off-target effects if antigens are expressed on normal tissues - Requires careful monitoring and supportive care including steroids	- Generally, well-tolerated but can cause immune-related adverse events (irAEs) such as colitis, dermatitis, and endocrinopathies - Side effects are typically managed with corticosteroids and other immunosuppressive agents	- Generally, well-tolerated with mild side effects - Risk of autoimmune reactions, depending on the vaccine design - Close monitoring required for adverse reactions
Patient Selection	- Suitable for patients with tumors expressing specific antigens and those who can tolerate the intensive treatment regimen - Requires careful patient selection to identify those most likely to benefit	- Suitable for a broad range of patients with tumors that express PD-L1 or have high mutational burden - Biomarker testing (e.g., PD-L1 expression, MSI status) can help identify patients likely to respond	- Suitable for patients with tumors expressing vaccine-targeted antigens. - Requires identification of appropriate antigens for each patient. - Potential for use in early-stage and high-risk patients.
Effectiveness	Highly effective at directly targeting and destroying tumor cells by recognizing specific antigens. They can be precisely tailored to target the unique markers of each tumor	Effective in reactivating exhausted T cells and enhancing the overall immune response, particularly in tumors with high PD-L1 expression.	- Efficacy varies; can induce durable immune responses and tumor control. - Often used in combination with other therapies for enhanced effectiveness.
Challenges	- Identifying appropriate antigens in benign tumors. - Managing potential side effects such as off- target effects and cytokine release syndrome (CRS). - Ensuring the persistence and activity of TCR/CAR-T cells in the tumor microenvironment.	- Overcoming resistance in tumors with low PD-L1 or CTLA-4 expression. - Managing autoimmune side effects due to broad T-cell activation. - Effectiveness can vary across different tumor types and patients.	- Identifying immunogenic and specific tumor antigens. - Ensuring robust and long-lasting immune responses. - Overcoming immune evasion mechanisms by tumors.
Current Research and Future Directions	- Exploring new antigen targets in benign tumors. - Enhancing T-cell engineering for improved specificity and reduced side effects. - Combining TCR/CAR-T therapies with other treatments for synergistic effects.	- Developing combination therapies with other immunotherapies. - Investigating biomarkers for better patient selection and treatment outcomes. - Expanding our understanding of checkpoint pathways in benign tumors.	- Developing next-generation vaccines with enhanced adjuvants. - Personalizing vaccines based on individual tumor antigens. - Combining vaccines with other immunotherapies and conventional treatments.

### Meningiomas

Meningiomas (**Figure 1D**) form from the arachnoid cap and meningothelial cells originating from neural crest tissue (**Table 1**) (26). They are classified as Grade I (benign), Grade II (atypical), or Grade III (anaplastic), wherein approximately 90% are benign (81). *NF2*, regulating contact-inhibited cell growth, is mutated in approximately 49% of all meningiomas. Other affected genes can include *TRAF7, AKT1, KLF4, POLR2A*, chromatin genes *KDM6A, CHD2*, and *SMARCB1* and tumor suppressor *PTEN*. Meningiomas segregate into four groups according to integrated molecular data (29) providing superior prediction of recurrence-free survival over WHO classification.

Chromosomal instabilities and genomic abnormalities can lead to increased metabolic aggressiveness (82). The complex immune environment of meningiomas involves mature memory/effector T and B cells, regulatory T cells, and cells expressing immune checkpoint molecules (83), indicating an opportunity for immune-based therapies (84). Adoptive T cell transfer targeting WT1 (**Table 2**) holds potential for treating skull base malignant meningiomas, revealing efficacy in mice and providing a promising approach for such challenging cases (77). Overexpressed WT-1 could be targeted vaccines to treat meningiomas (60). A major prognostic factor in low-grade meningiomas involves immune infiltration by dendritic cells and M2 macrophages, and CSF-1R inhibitors to decrease M2 macrophage populations or targeting MDSCs with IDO- or arginase inhibitors may further improve meningioma immune responses (78).

### Nevi

Congenital melanocytic nevi (CMN)(moles), consist of benign proliferating melanocytes protruding into the deep dermis. Nevi are classified by size, shape, and color. Large and giant CMN are associated with an increased risk of developing melanoma and neurocutaneous melanosis (NCM) (33) NCM is caused by rare proliferation of melanocytes within the CNS. Immunotherapies can well serve to treat recurrent, premalignant nevi, large neurocutaneous melanosis, and large congenital melanocytic nevi to avert the risk of progression (32).

Acquired nevi (**Figure 1E**) often harbor *BRAF* mutations linked to aging and UV exposure, whereas CMN typically exhibit *NRAS* mutations (**Table 1**), indicating a distinct molecular basis. While *BRAF* and *NRAS* mutations are mutually exclusive, both activate the MAPK pathway to drive melanocytic neoplasia (32). Somatic mutations in codon 61 of *NRAS* are prevalent among CMN and NCM (33). Immunotherapy can induce regression, often by mechanisms shared with melanoma treatment. Herein immune checkpoint inhibitors (**Table 3**) including anti-CTLA-4 enhance T cell activation, mediating regression of atypical nevi and a vitiliginous reaction (62). *NRAS*-mutated melanomas exhibit improved clinical responses and progression-free survival over *BRAF*-mutated melanomas after IL-2 or checkpoint inhibitor therapy (79). These findings pave the way for immunotherapy targeting nevi.

Melanocytic nevi express melanosomal proteins including MART-1, tyrosinase, and gp100 as potential targets for adoptive cell transfer or vaccine-based treatments (34). MART-1-specific CTLs (**Table 2**) can regress both benign nevi and melanoma lesions in an antigen-specific manner (61). Nevus-resident type 1 CD4+ T cells also rejected benign nevi in an antigen-specific manner, highlighting the use for melanosomal antigens as effective immunotherapy targets (63). The target molecules in nevi are shared exclusively with melanocytes, and are predominantly melanosomal in nature. This selectivity would be advantageous for immunotherapies (62).

### Neuromas

#### Symptomatic neuromas

Neuromas (**Figure 1F**) in the PNS arise from failed tissue repair and abnormal cell growth after nerve damage, occurring in 5-10% of nerve injury patients and more in individuals undergoing amputations (35). By exception, most neuromas are not associated with mutations, yet the predictable pathophysiology of symptomatic neuromas can provide opportunities for immune targeting. Neuromas present as disorganized nerve fiber tangles encapsulated in fibrous tissue. Nerve myelination is greatly reduced with evidence of chronic inflammation caused by infiltrating mast cells, proliferating fibroblasts and glycosaminoglycan rich stromatic tissue. SMA^+^ myofibroblasts are overabundant. Secretion of neuropeptides like CGRP and pro-inflammatory cytokines is upregulated (37). M1 Macrophages are recruited, exacerbating the pain response (85). Treatment with anti-inflammatories might reduce pain and neuroma sizes (38, 39). Modulating the immune environment can remove proinflammatory molecules from the blood, and have shown potential in attenuating disease progression.

#### Vestibular schwannomas (acoustic neuromas)

Vestibular schwannomas (VS) are benign growth of Schwann cells on the vestibular portion of the eighth cranial nerve (86). While 95% of VS are sporadic, 5% are associated with mutations in *NF2* encoding merlin, a tumor suppressor protein related to the ERM (ezrin-radixin-moesin) family (87). Neurofibromatosis covers multiple tumor types, including acoustic neuromas. Alternate types include mutations in NF1 which are more susceptible to malignant transformation. The epidermal growth factor receptor HER1 was found to be overexpressed in the latter tumor type, prompting the development of immunosuppression-resistant CAR T-cells that displayed anti-tumor efficacy *in vitro* and in tumor spheroids (88). Its loss from Schwann cells dysregulates signaling pathways such as PI3k-Akt, Wnt, Hippo-YAP/TAZ mTORC1 and EGFR and stimulates Schwann cell growth and migration (89). Upon de-differentiation, Schwannoma cells promote nerve repair and immune cell recruitment (90). marked by elevated cytokines that drive tumor progression, immunosuppression, and stroma formation (40). Recruited immune cells include macrophages, T-lymphocytes including regulatory T cells and NK cells (91).

The tumor microenvironment contributes to VS pathogenesis, and offers potential therapeutic targets (92). VSIG4 is expressed on M2 macrophages, inhibiting CD8+ T cell cytotoxicity after binding to ligands (92). Treatment with VISG4 antibodies reduced disease severity in a mouse model of experimental autoimmune encephalomyelitis, and might serve schwannoma treatment as well (42). Angiogenesis plays a key role in VS lesion progression (41). Treatment with an anti- vascular endothelial growth factor (VEGF) antibody reduced tumor burden and improved the hearing of NF2 patients (93). VS tumor cells expressed high levels of VEGFR1 and VEGFR2, and VEGF receptor peptide vaccination in NF2 vestibular schwannomas also demonstrated signs of intratumoral apoptosis (64).

### Osteochondromas

Osteochondromas (OC) (**Figure 1G**) develop as bony outgrowths with a cartilage cap projecting from the bone surface, typically located in the metaphysis of long bones (45). Most are solitary lesions, while 15% of cases are associated with hereditary multiple osteochondroma (HMO), an autosomal dominant disorder (94). Malignant transformation occurs in 1% of solitary and 10% of HMO cases. Recurrence is observed in approximately 5% of sporadic and 20% of HMO osteochondroma patients, dropping to less than 2% if complete resection is achieved (44). Pathogenic variants in *EXT1* or *EXT2* were detected in 85% of HMO cases and 80% of solitary cases (46). The genes encode enzymes critical for heparan sulfate biosynthesis, important for cell differentiation and tissue morphogenesis. Heparan sulfate binds growth factors and extracellular matrix proteins, and supports leukocyte migration and recruitment (95). High EXT1 expression is associated with an abundance of CD8+ T cells, while EXT2 expression is associated with reduced CD4+ T cells, macrophages, neutrophils, and dendritic cells in head and neck squamous cell carcinoma (HNSC) (96).

ERK signaling suppresses chondrocyte proliferation, lineage commitment and differentiation, while its loss in CD4+ cells leads to osteochondroma-like structures lacking immune cell infiltration in mice (97). T cell loss accelerated tumor growth. A patient with biallelic germline mutations in Nuclear Factor of Activated T Cells-2 (*NFATC2*) presented clinically with recurrent B cell lymphoma and osteochondromas (65). *NFATC2* encodes the NFAT1 transcription factor which responds to calcium signaling and controls gene expression in immune cells and chondrocytes. NFAT1-deficient CD4+ T cells expressed elevated PD-1 and reduced TNF-*α* and IFN-*γ*, underscoring a role for the immune system (65). There would be great benefit to developing osteochondroma immunotherapies such as checkpoint inhibitors, which restore T-cell activation and enhance anti-tumor immunity by inhibiting immune checkpoint signaling.

### Condylomas

Condylomas (**Figure 1H**) are benign epithelial growths often caused by human papillomavirus (HPV) types 6 and 11 (49). HPV is a significant global health issue, with a prevalence of 11–12% or more for oncogenic types 16 and 18 in females regardless of cervical abnormalities (47). Preventive vaccines are highly effective against the main HPV types responsible for benign genital warts and cervical malignancies (48).

The HPV6 E5 protein decreases MHC class 1 expression, rendering infected cells less detectable to cytotoxic T cells (98). Clinical HPV vaccine trials, particularly those targeting precancerous papillomas, have shown great promise (99). Trials with therapeutic mRNA-based and nanoparticle vaccines targeting the HPV E7 protein likewise revealed promising outcomes (51). Immune checkpoint proteins, growth factors, and immunoregulatory proteins are expressed within the papilloma microenvironment, highlighting a complex immunoregulatory landscape (100). Overall, the development of HPV vaccines offers promise for the treatment for premalignant HPV lesions.

### Lipomas

Lipomas (**Figure 1I**) are soft tissue tumors characterized by adipocyteproliferation and they often arise in the subcutaneous tissue (52). In 5–15% of cases, patients present with multiple lipomas, which may occur sporadically (e.g. Dercum’s disease) or result from inherited genetic abnormalities such as PTEN hamartoma syndromes (e.g. Cowden syndrome) and neurofibromatosis type 1 (52). Dercum’s disease, a rare inflammatory condition characterized by painful subcutaneous fat masses and alterations in lymphatic vessels, represents a promising indication for immunotherapy due to its underlying immune dysregulation and inflammatory nature (53).

Deoxycholic Acid injections triggered adipose degradation by macrophages, tumor shrinkage and pain mitigation (101). Emerging research suggests potential immunotherapeutic targets within the lipoma microenvironment. Macrophage polarization, particularly the predominance of M2 macrophages, may contribute to lipoma persistence, suggesting that CSF1R inhibitors could shift the immune response toward M1 activation to promote adipocyte apoptosis (102). Additionally, immune checkpoint inhibitors such as PD-1/PD-L1 blockade (pembrolizumab, nivolumab) may enhance T-cell activity if lipomas exhibit immune evasion mechanisms. Lipomas are associated with gene rearrangements involving, for example, lipoma preferred protein (LPP) or HMGA2. Tumor-associated overexpression of the resulting proteins provides a rationale for the design of peptide vaccines to promote immune targeting. The potential efficacy of immune-based approaches for lipomas is evidenced by the remarkable tumor shrinkage observed in a patient treated by CD19 CAR therapy (55).

## Discussion

Benign tumors, perceived as less severe or life-threatening than malignancies, have historically been overlooked for therapeutic innovation. However, these tumors can cause debilitating symptoms that profoundly impact patient quality of life and lead to life-threatening complications. Through our analysis of the latest research, clinical outcomes, and existing therapies, we identified promising opportunities to explore immunotherapies as transformative treatments for benign tumors, some of which are already being put to the test.

Sporadic examples of immunotherapeutics used for the treatment of benign tumors *in vivo* include Garzon-Muvdi et al. (103) who reviewed ICI as an option for meningiomas, combined or not with NK cells in preclinical models. Azab et al., 2023 (104), also made a case for meningiomas, describing MDSCs as tumor drivers and showing that infiltration by relevant T cells and PD1 and PD-L1 expression are reliable prognostic predictors, while discussing mutations that could provide neoantigens for infiltrating T cells. IFNα has been among the earlier proposed immunotherapeutics tested for meningiomas, with some efficacy (105). Pituitary adenomas have also been the subject of preclinical studies, where animal models of Cushing’s disease and clinical studies did reveal survival benefit and treatment efficacy (106) from checkpoint inhibitor therapy. In a patient with laryngeal papillomas, the IL-5 receptor on eosinophils proved a target for antibody therapy (107). Halkola et al. (108) favored anti-angiogenic treatment for benign tumors, following suggestions of the same by Hannan et al. (109), though the treatment not only limits oxygen access but also access to immune cells. In patients with HNSCC, benign tumors provided a greater opportunity to propagate cytotoxic NKT cells than malignant ones (110). Warts were successfully injected with skin test antigens to induce T cell infiltration (111). Candida antigen has been used in this respect (56). Immune stimulation with imiquimod +/- green tea derivatives has shown some benefit (112). Hyperthermia was an early treatment to evoke immune responses, supportive of tumor regression in cows (113).

In solid organ transplant patients, BCG treatment did not temper the risk for developing bladder tumor (114), this outcome is potentially related to immunosuppressive treatment provided to this population. Patients might benefit from ICI and other less specific treatments, yet we promote the concept that knowledge of benign tumor physiology offers an opportunity to direct immune responses to the tumors, for greater specificity and efficacy. Our own studies showed marked efficacy of GD3 CAR T cells against benign tumors modeling TSC (3). Likewise, gp100/PMEL proved a credible target for T cells in lymphangioleiomyomatosis (LAM) (115). We subsequently showed efficacy for adoptive transfer of gp100/Pmel-reactive T cells for the condition in preclinical studies (116).

Moreover, Liu et al. (117) have shown that in preclinical mouse studies, CTLA-4 blockade combined with anti-PD1 provided greater tumor clearance of TSC-associated tumors than anti-PD1 treatment alone. The paper further describes a supportive mechanism for this observation by demonstrating increased T cell infiltration accompanied by elevated IFNγ and TNFα production, potentially overcoming B7-H3-mediated immune evasion reported in LAM (118). This work supports the very real potential of checkpoint inhibition for the treatment of benign tumors, which might be further enhanced when including antigen specificity to the treatment regimen.

When considering immunotherapeutic strategies, it will be important to understand if these tumors form as part of a hereditary syndrome or as a consequence of *de novo* mutations only, to understand the likelihood of the mutated gene product or its downstream effectors to be recognized by the patient’s immune system. In this respect, patients with hereditary tumor syndromes might draw specific benefit from immunotherapeutic approaches, if recurring tumors can be eliminated by T cell recall responses. The observed persistence of adoptively transferred CAR T-cells holds specific significance for these patients (19). Another consideration is the potential for side effects. ICIs for example can elicit adverse events ranging from skin rash to gastric or cardiac events; including an antigenic vaccine can provide direction to the immune response and limit such side effects, but such vaccines, in turn, deserve some scrutiny to understand if the epitopes included might be shared with homologous tissue antigens or with expression outside the tumor itself and could incite on target, of tumor responses that would do harm. These risks have formed the greatest impediment to applying immunotherapy for the treatment of benign tumors until the present day. This is not different from any other therapeutic application used to treat the condition, and with caution and consideration for potential consequences, the benefit of immunotherapy can well outweigh the risks.

Methods traditionally successful in treating malignant tumors like adoptive T-cell transfer, checkpoint inhibitors, or tumor vaccines (**Table 3**) could be beneficial. Immunotherapy strategies for benign tumors focus on preventing tumor progression, recurrence, or malignant transformation by leveraging immune modulation. Vaccination can be used if the benign tumor expresses new antigens, training the immune system to recognize and eliminate abnormal cells early. Varying the mode of application (e.g., peptide vaccines, mRNA, dendritic cells) enhances immune activation and prevents tolerance. Anti-CTLA-4 therapy (e.g., ipilimumab) promotes antigen-specific T cell responses, while anti-PD-1 inhibitors (e.g., pembrolizumab) sustain immune surveillance by preventing T cell exhaustion; other targets can finetune the outcomes. In cases requiring more targeted intervention, adoptive immunotherapy with transgenic T cells (e.g., CAR-T or TCR-T cells) can selectively eliminate tumor cells while preserving healthy tissue. This immunotherapeutic approach aims to control benign tumors, reduce recurrence, and prevent malignant progression. While immunotherapeutics have faced challenges in the clinic, the less aggressive nature and more predictable biology of benign tumors may create more favorable conditions for success. Furthermore, identifying tumor specific antigens has become more feasible as deeper genomic, phenotypic, and molecular data of these benign tumors become available. In conclusion, immunotherapeutics hold great promise for treating benign tumors. In future clinical trials, these concepts can be validated to establish new standards of care, ultimately enhancing the management of benign tumors.
